# Supplementary material for: Impact of Experimental Bias on Compositional Analysis of Microbiome Data
Source: Genes (Basel). 2023 Sep 8;14(9):1777. doi: 10.3390/genes14091777 (PMC10530728; doi:10.3390/genes14091777)
Supplement: Supplementary file 1 [file genes-14-01777-s001.zip › genes-2570145-supplementary.pdf]

Supporting Information

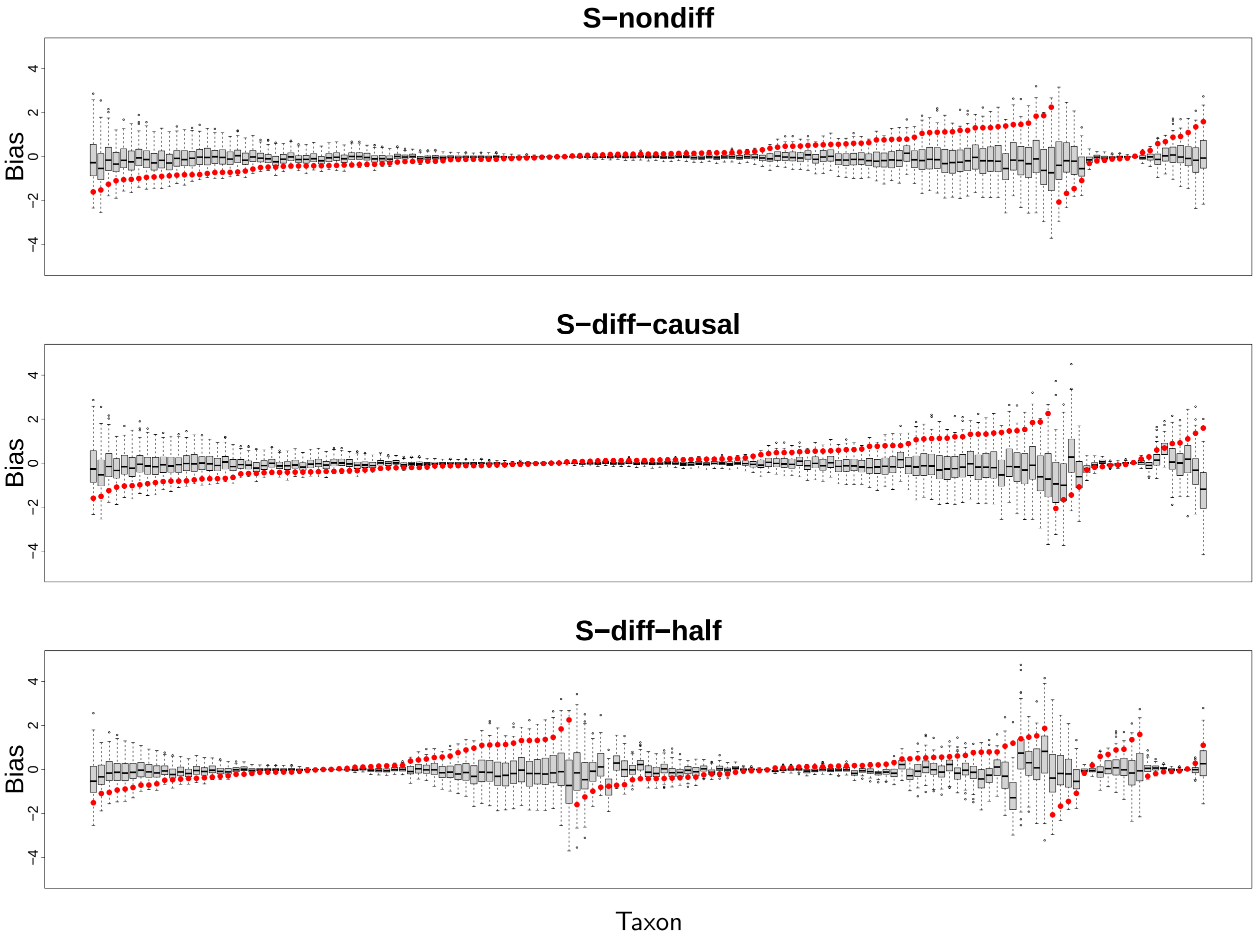

Figure S1. Distribution of the bias factor due to taxon-taxon interactions, $\eta_{ij} = \sum_{j' \neq j} \theta_{jj'} \pi_{ij'}$, based on one replicate of data that were generated under M1 with $\exp(\beta) = 5$ and $\phi = 4$ and had a binary trait. Each box plot represents the distribution of η_{ij} in taxon j across all samples. Each red dot represents the taxon-specific main bias factor γ_j for taxon j. Only taxa that passed our filter are shown. In each panel, we sorted the taxa so that the null taxa appeared first and the causal taxa next, and then, within each group (null or causal), we sorted the taxa in ascending order of the main bias factor. In S-diff-half, we additionally sorted the taxa within each group so that the taxa belonging to the one half of taxa (with $\epsilon_{jj'}/2$ sampled from $N(0.5, 0.1^2)$) appeared first and the taxa belonging to the remaining half of taxa (with $\epsilon_{jj'}/2$ sampled from $Beta(0.5, 0.5)$) appeared next.

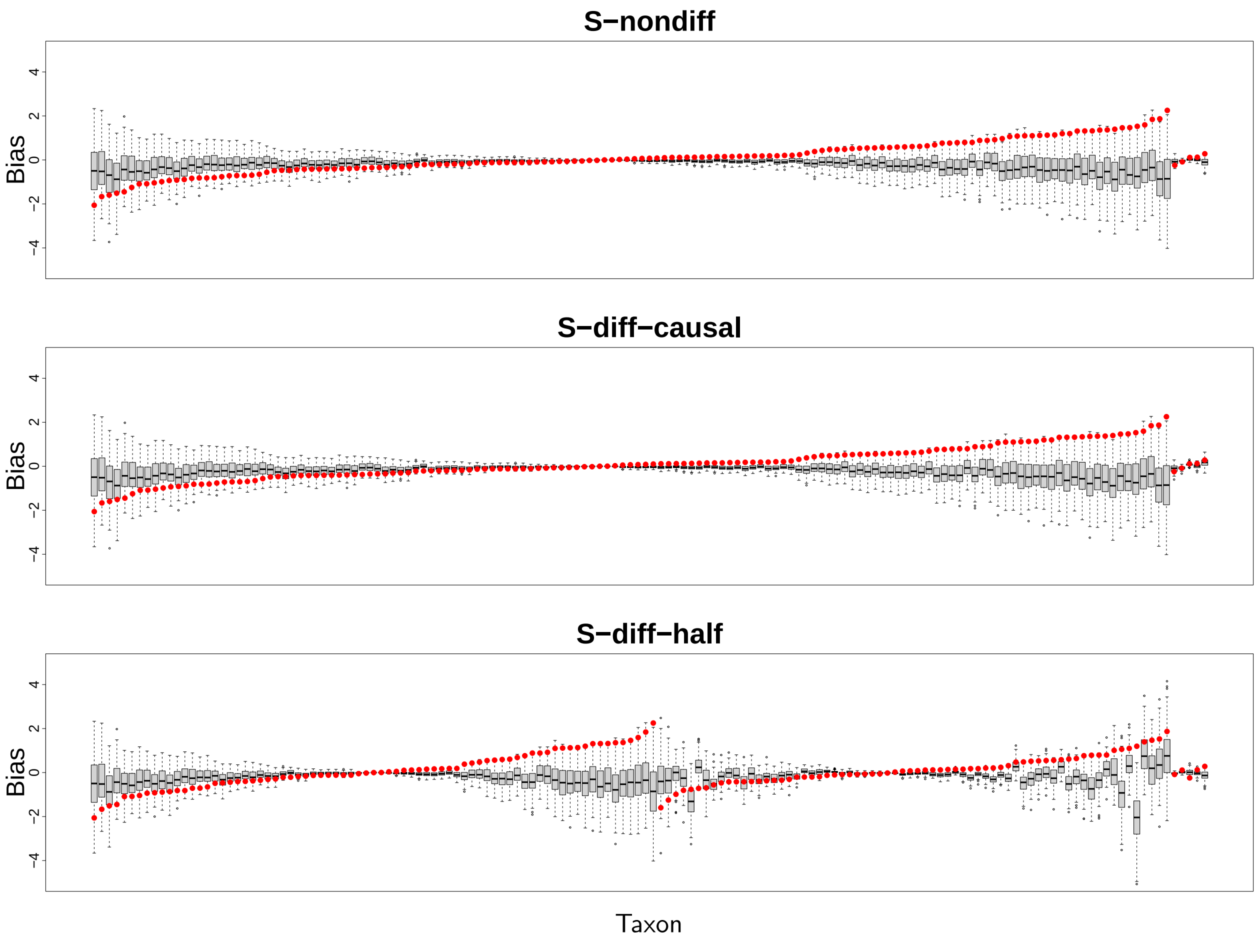

Figure S2. Distribution of the bias factor due to taxon-taxon interactions, based on one replicate of data that were generated under M2 with $\exp(\beta) = 3$ and $\phi = 4$ and had a binary trait. See more information in the caption of Figure S1.

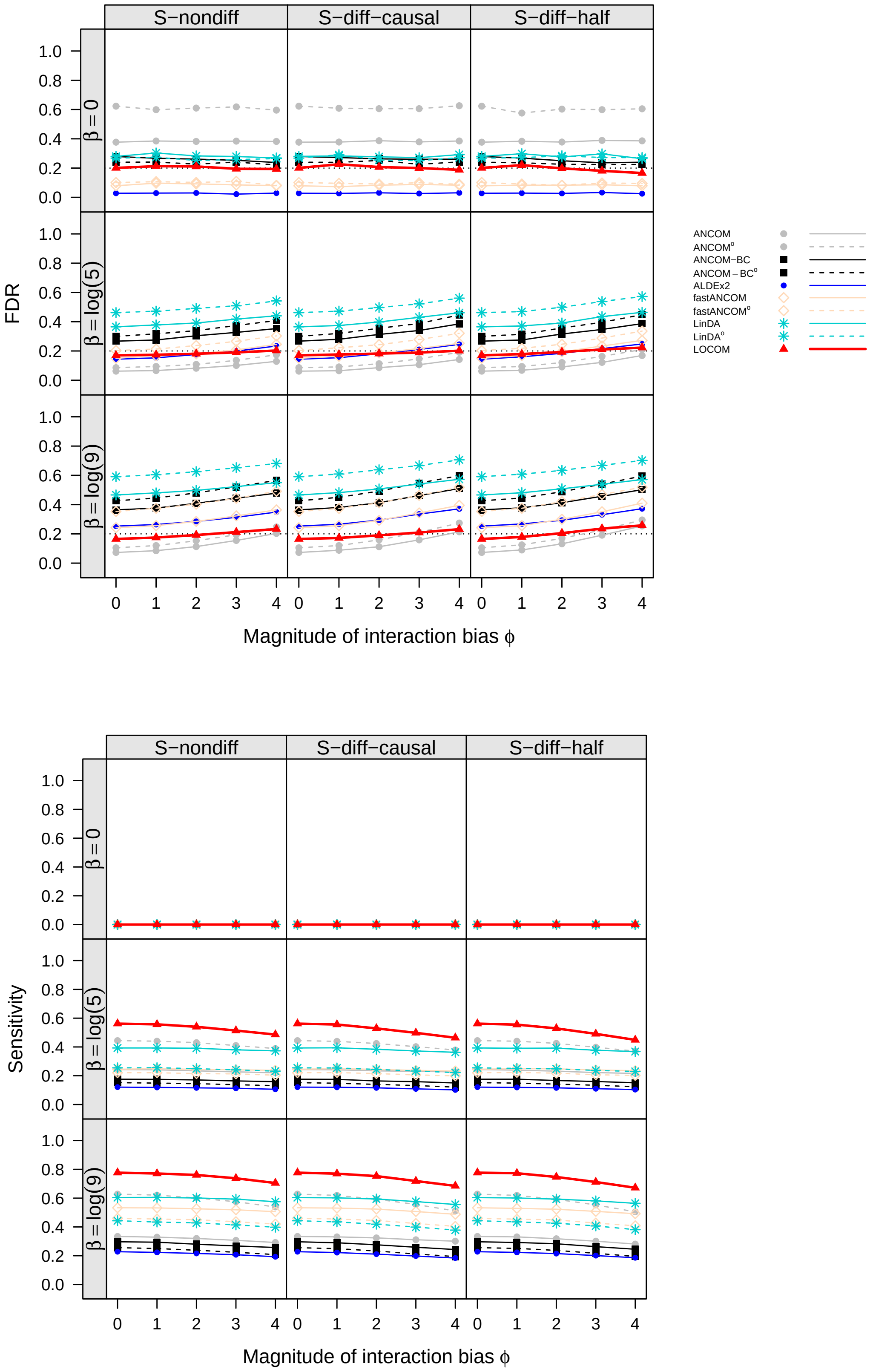

Figure S3. Similar to Figure 2 except that the data were simulated under M1 and had a binary trait and a binary confounder.

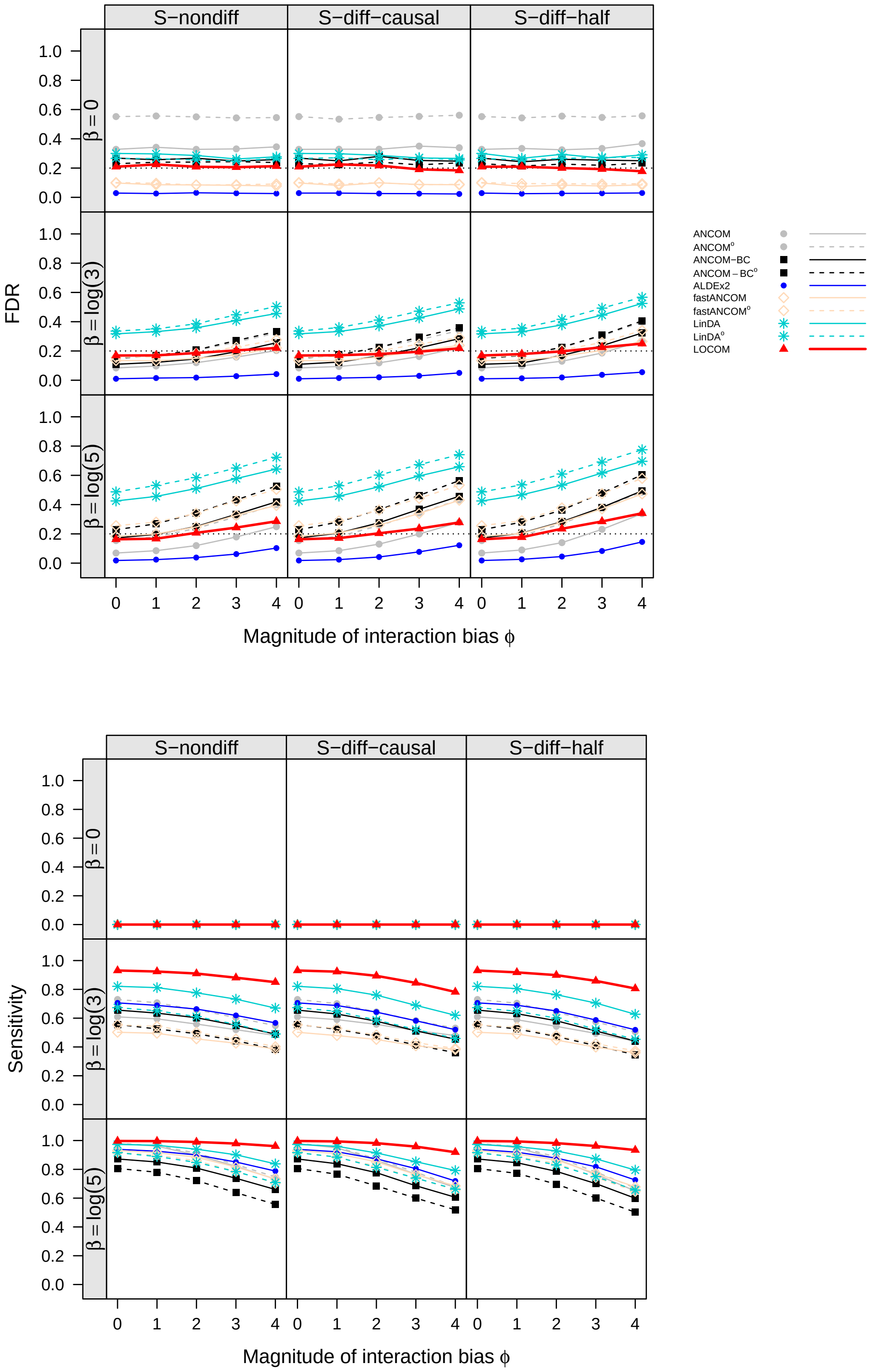

Figure S4. Similar to Figure 2 except that the data were simulated under M2 and had a binary trait and a binary confounder.

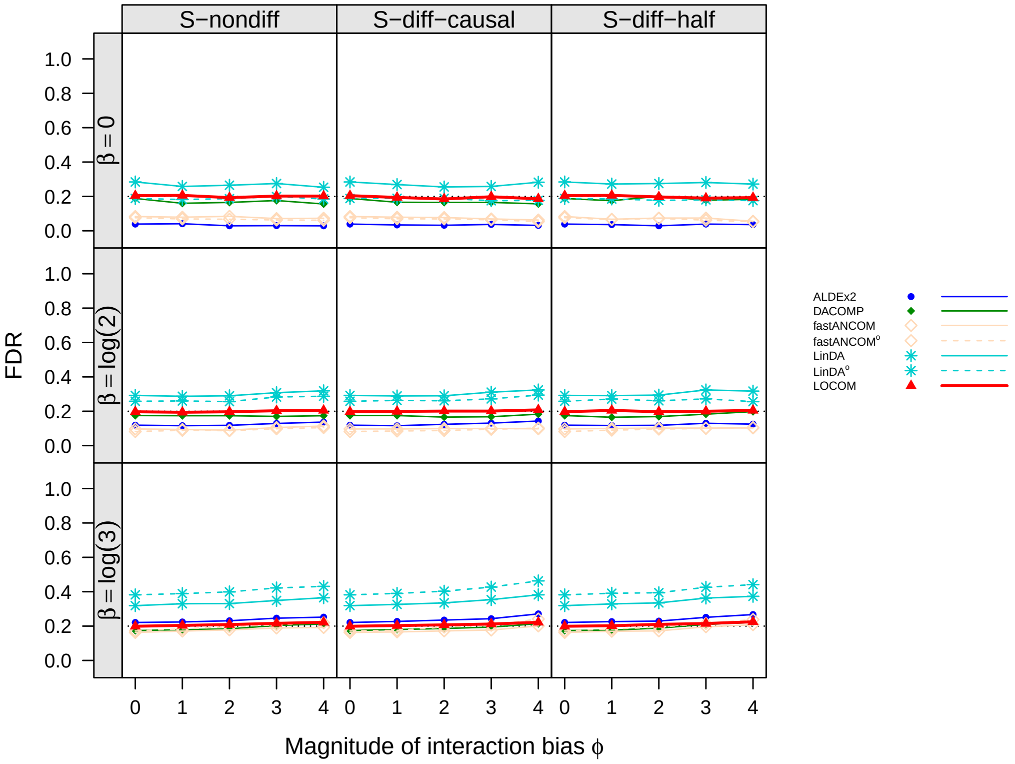

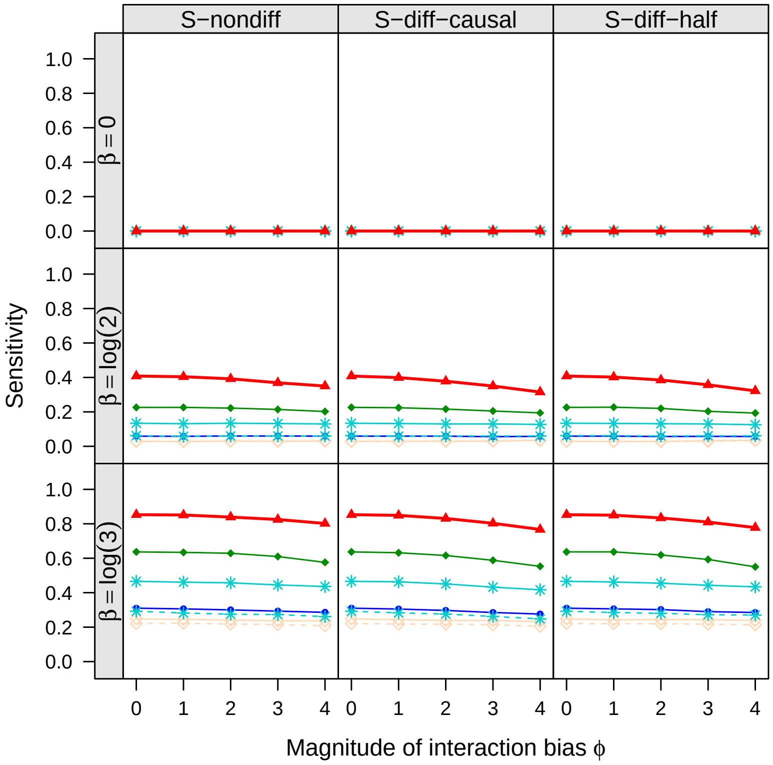

Figure S5. Similar to Figure 2 except that the data were simulated under M1 and had a continuous trait.

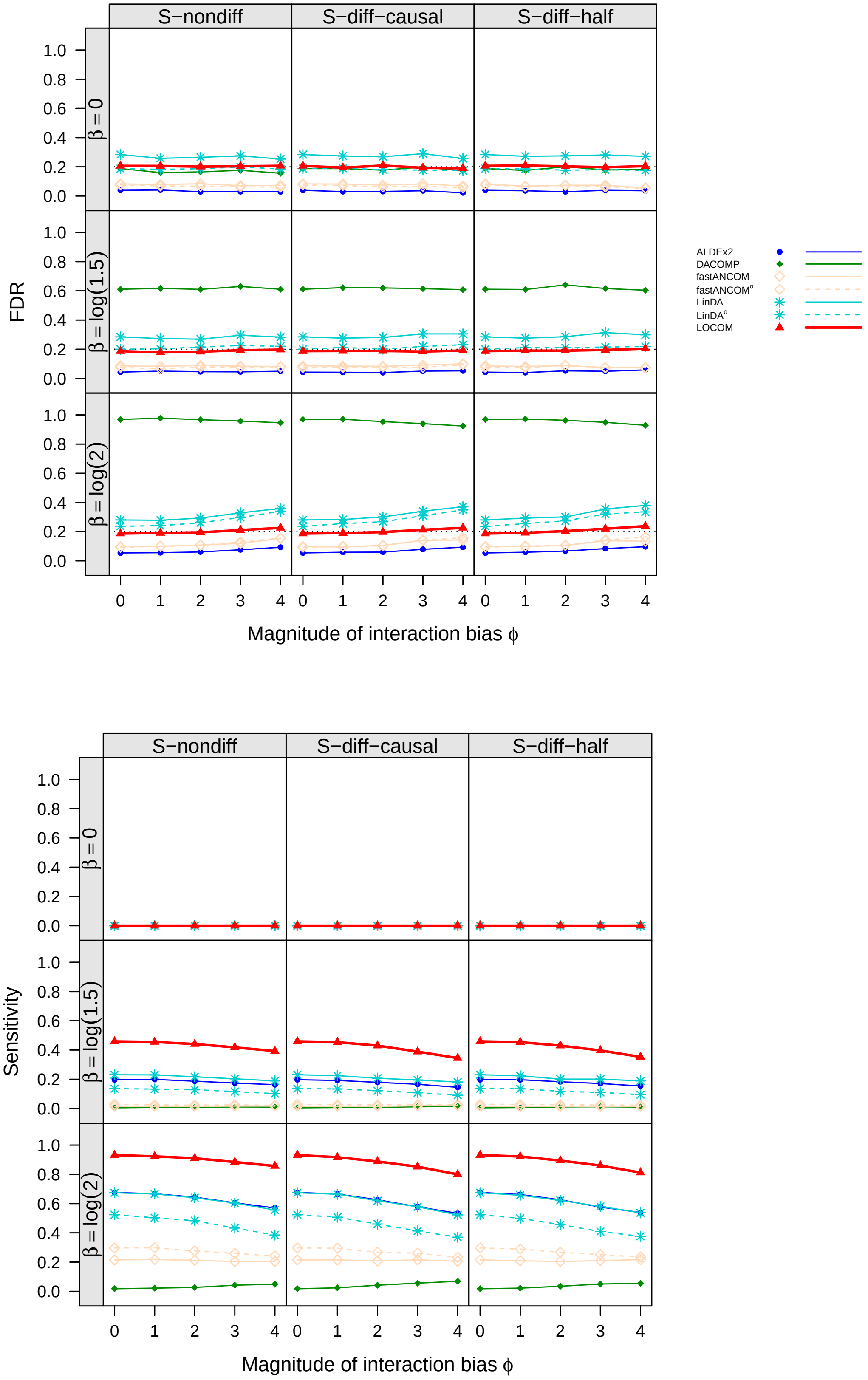

Figure S6. Similar to Figure 2 except that the data were simulated under M2 and had a continuous trait.
